# A two-stream convolutional neural network for microRNA transcription start site feature integration and identification

**DOI:** 10.1038/s41598-021-85173-x

**Published:** 2021-03-11

**Authors:** Mingyu Cha, Hansi Zheng, Amlan Talukder, Clayton Barham, Xiaoman Li, Haiyan Hu

**Affiliations:** 1 Department of Computer Science, University of Central Florida, Orlando, FL USA; 2 Burnett School of Biomedical Science, College of Medicine, University of Central Florida, Orlando, FL USA

**Keywords:** Machine learning, Genomics

## Abstract

MicroRNAs (miRNAs) play important roles in post-transcriptional gene regulation and phenotype development. Understanding the regulation of miRNA genes is critical to understand gene regulation. One of the challenges to study miRNA gene regulation is the lack of condition-specific annotation of miRNA transcription start sites (TSSs). Unlike protein-coding genes, miRNA TSSs can be tens of thousands of nucleotides away from the precursor miRNAs and they are hard to be detected by conventional RNA-Seq experiments. A number of studies have been attempted to computationally predict miRNA TSSs. However, high-resolution condition-specific miRNA TSS prediction remains a challenging problem. Recently, deep learning models have been successfully applied to various bioinformatics problems but have not been effectively created for condition-specific miRNA TSS prediction. Here we created a two-stream deep learning model called D-miRT for computational prediction of condition-specific miRNA TSSs (http://hulab.ucf.edu/research/projects/DmiRT/). D-miRT is a natural fit for the integration of low-resolution epigenetic features (DNase-Seq and histone modification data) and high-resolution sequence features. Compared with alternative computational models on different sets of training data, D-miRT outperformed all baseline models and demonstrated high accuracy for condition-specific miRNA TSS prediction tasks. Comparing with the most recent approaches on cell-specific miRNA TSS identification using cell lines that were unseen to the model training processes, D-miRT also showed superior performance.

## Introduction

MicroRNAs (miRNAs) are ~ 22 nucleotides long non-coding RNAs. They express ubiquitously in almost all cell types, are evolutionarily conserved in most metazoan and plant species, and can regulate more than 30% of mammalian gene products through complementary binding to the corresponding mRNAs^1,2^. MiRNAs have been shown to participate in various types of biological processes such as cell development, differentiation, and apoptosis. Misexpression of miRNAs has been linked to complex diseases such as cancer, diabetes, and heart disease^3–5^. Therefore, understanding how miRNAs are regulated under different conditions is critical to understand gene regulation and disease phenotypes.

Transcription start sites (TSSs) are the key to define promoter regions and understand gene regulation. In contrast to our knowledge of TSSs of protein-coding genes, much less is known about the locations of TSSs for miRNA genes under different cellular conditions. MiRNA TSSs can be quite far away (e.g. 10 kilobase pairs (kbp)) from the corresponding mature miRNAs due to the specific process of miRNA biogenesis^6–8^. During the process, long primary miRNAs (pri-miRNAs) are first transcribed. These pri-miRNAs are subsequently processed by a microprocessor consisting of the nuclear RNase III Drosha and its cofactor protein DGCR8 to generate precursor miRNAs (pre-miRNAs). Cleavage of the generated pre-miRNAs by another RNase III protein Dicer produces small RNA duplexes, which form RNA-induced silencing complexes (RISCs) together with AGO proteins and generate the mature miRNAs. MiRNA biogenesis is so transient that regular RNA-Seq experiments cannot capture a sufficient amount of pri-miRNAs, and as a consequence, identifying the TSSs of miRNA genes is more difficult than identifying the TSSs of protein-coding genes.

Initial computational approaches for genome-wide TSS identification focused on the use of sequence features such as over-represented k-mers, transcription factor binding site enrichment, conservation, and CpG content^9–13^. Such sequence-based strategies often lead to a large number of false predictions and are not able to identify condition-specific TSSs. Later studies have shown success in miRNA TSS prediction utilizing active gene transcription markers such as trimethylation of Lys 4 of histone 3 (H3K4me3), acetylation of Lys 9/14 of histone 3(H3K9/14Ac), Polymerase II (Pol II) and DNase-Seq measurements^14–18^. In the past decade, TSS-Seq technologies such as Cap Analysis Gene Expression (CAGE) have enabled the genome-scale detection of transcription initiation events^19,20^. With TSS-Seq data available in many tissues and cell lines, computational methods have been created to identify miRNA TSSs through integrative analysis of TSS-Seq data and epigenetic markers^21–23^. For example, miRStart was developed to model miRNA TSSs with a Support Vector Machine (SVM) integrating 29 million CAGE tags from the FANTOM4 project, nearly 1 billion TSS-Seq data points, and H3K4me3 chromatin signatures in human CD4 + T cells^21^. This approach led to the prediction of 847 miRNA TSSs. PROmiRNA integrated FANTOM4 CAGE data and sequence features such as conservation and CpG content to identify true miRNA TSS regions from background genomic regions based on an Expectation–Maximization algorithm^22^. However, pooling epigenetic marker information from different cell lines, these methods do not aim for condition-specific miRNA TSS identification.

A couple of most recent work attempted to identify condition-specific miRNA TSSs. For example, taking advantage of deeply sequenced RNA-Seq data and also integrating epigenetic markers in a condition-specific manner, microTSS was able to make high-resolution predictions^7^. Hua et al. created an alternative approach that does not require deeply-sequenced RNA-Seq data, which was able to identify condition-specific miRNA TSSs corresponding to 54 cell lines in the ENCODE project using sequence conservation measurements, H3K4me3 and DNase-Seq information^24^. Instead of using CAGE TSS-Seq data, mirSTP directly extract miRNA TSSs from global/precision nuclear run-on RNA-Seq data (GRO-Seq) that are available for specific cellular conditions and use a probabilistic model to predict miRNA TSSs^25^. These existing computational methods have predicted a large number of miRNA TSSs and provided insights into miRNA gene regulation. However, high-resolution condition-specific miRNA TSS annotation remains a challenging problem due to the lack of understanding of miRNA TSS features^26^. MiRNA TSS prediction algorithms from different studies often use different feature sets as predictors and their results are often inconsistent with each other^18^. Besides, most of the recent miRNA TSS predictions using epigenetic markers such as H3K4me3 profiles often lead to low-resolution predictions essentially due to the low-resolution properties of these markers. In terms of modeling algorithms, SVMs are the most commonly used models in literature for the miRNA TSS prediction task^7,21^, whereas deep learning models using multi-layered neural networks for modeling and prediction are the most recent development in machine learning field^27^. In fact, deep learning algorithms are capable of automatically extracting useful features and has recently shown superior performance in various types of bioinformatics problems compared to SVMs^28^. So far, deep learning based algorithms have not been tested on the specific problem of condition-specific miRNA TSS prediction.

This study designed a two-stream deep neural network model to integrate features at varied resolution levels towards miRNA TSS identification, abbreviated as D-miRT (http://hulab.ucf.edu/research/projects/DmiRT/). D-miRT is structured as a two-component deep Convolution Neural Network (CNN). The first component is a CNN that is responsible for learning from low-resolution features, for example, epigenetic markers such as ChIP-Seq peak profiles of H3K4me3 and DNase-seq. In contrast, the second component is a CNN responsible for learning from high-resolution features, for example, single-base resolution nucleotides in input sequences. Unlike previous studies that focused only on sequence features that cannot detect cell-specific TSSs, or only on TSS-related chromatin features that lead to low-level resolution predictions, D-miRT is able to take into account different types of features and integrate them using a deep-learning framework. D-miRT also selects training and testing data from the 1357 human miRNA promoter regions under multiple cell conditions recently identified by the FANTOM5 project^29^. These newly annotated miRNA TSSs provide a relatively sufficient data set for training and benchmarking miRNA TSS predictions. By integrating both sequence features and features from high-throughput sequencing datasets, D-miRT has shown the ability to produce accurate, high-resolution, and condition-specific miRNA TSS predictions.

## Materials and methods

### Human data collection

About 1900 miRNAs and their genomic coordinates were downloaded from the miRBase22 database and converted from hg38 to hg19 using CrossMap0.3.3^30^. The recently available 1,357 miRNA promoters were obtained from FANTOM5^29^. The protein-coding gene TSSs were downloaded from GENCODE v32^31^. Seven cell lines GM12878, K562, HeLa-S3, HepG2, MCF7, A549 and hESC common to both FANTOM and ENCODE projects were selected for model training and/or evaluation. CAGE robust TSS peaks with coverage in transcripts per kilobase million greater than 10, for these seven cell lines were downloaded from the FANTOM5 project. H3K4me3 and DNase-Seq peak data for the seven cell lines were downloaded from the ENCODE project^32^. The complete human genome, together with its phastCons 46-way placental conservation score, was taken from the UCSC Genome Browser website^33^ (Supplementary Table S1).

### Data processing

**Figure 1:**
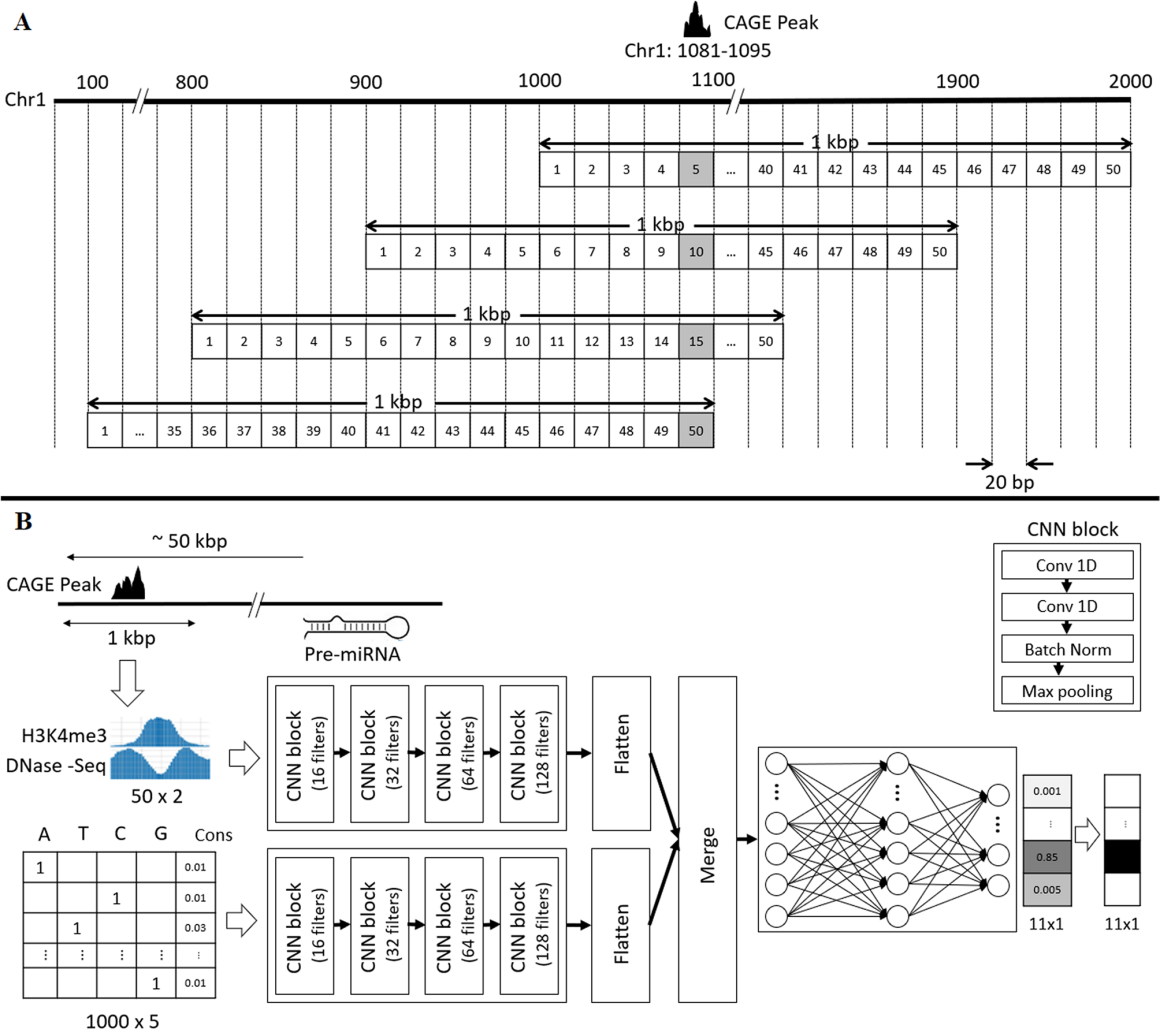
The D-miRT architecture. **(A)** The input matrix for the first CNN component and the output labels of D-miRT are created by sliding and binning the 1 kbp region around a CAGE peak. The bin number containing the TSS peak is used as training label for an input positive sample. **(B)** Two separate CNN components for activation marker and sequence inputs, each one having 4 CNN blocks. The features learned by the two components are merged and fed through a three-layer fully connected neural network that outputs the probability of a TSS to be within a certain TSS-containing bin.

Various types of TSS distribution patterns such as narrow peak and broad peak distributions for different cell lines have been identified so far^34,35^. The training and test data were generated using the narrow CAGE peaks from the FANTOM5 depository for the aforementioned seven cell lines. The CAGE peaks with 0 tag or wider than 10 base pairs (bp) were filtered out. The CAGE peaks that did not contain a protein-coding gene TSS were also filtered out. The final set of CAGE peaks were considered as “TSS peaks” for training the model. TSS peaks that occur within 1 kbp of each other were filtered out to reduce the data complexity. Every 1 kbp region containing a TSS peak was divided into 50 bins of size 20 bp each. Note that since a TSS peak width cannot be more than 10 bp and the bin size is 20 bp, every TSS peak resided in only one of the 50 bins (Fig. 1A). In order to train the model about the location of the TSS in the input region, for every TSS peak, 10 input samples were generated by sliding the 1 kbp region by 100 bp (5 bins) each time for 10 times. The 10 input samples (positive samples) are 1 kbp regions having the TSS peak in 10 different bins (Fig. 1A). In order to train the model about the input regions with no TSS, a 1 kbp flanking region of the TSS peak that did not contain any TSS peak was also included (negative samples). The output of the model is an 11 × 1 vector. The row numbers 0 to 10, correspond to the probabilities that a region is from the negatives or each of the 10 types of positives (Fig. 1B). Representation of the input and output in such a way enables the model to answer both the questions “is there a TSS?” and “where is the TSS?”.

In order to predict a TSS within a region, D-miRT considers both the H3K4me3 and DNase-Seq profiles and the sequence features within the region. For every sample, a 50 × 2 matrix is generated with the DNase-Seq and the H3K4me3 peak coverages in the 50 bins of the corresponding input region. As to the sequence features, the one-hot encoding of the sequence and its conservation score of every genomic location within the 1 kbp TSS-containing region were used to create a 1000 × 5 input matrix. The one-hot encoding is a 1 × 4 vector representation of a nucleotide ([1 0 0 0] for A, [0 1 0 0] for T, [0 0 1 0] for C and [0 0 0 1] for G). After binning and shifting the TSS-containing regions 10 times and including the negative regions without TSS, the number of samples generated for the seven cell lines A549, GM12878, HeLa-S3, HepG2, hESC, K562 and MCF-7 are 83,897, 108,988, 109,802, 113,751, 100,133, 96,965 and 101,838, respectively, including both the positive samples and negative samples.

### Deep-learning model

Deep Learning is a family of machine learning methods that are based on artificial neural networks. CNN is a class of deep neural networks that can capture local spatial patterns and use them to recognize higher-level features and patterns. Since commonly used epigenetic markers and sequence features exhibit local spatial patterns, CNN is a promising solution for the miRNA TSS prediction task. Therefore, a CNN-based model D-miRT was designed for miRNA TSS prediction. A CNN model typically consists of convolutional, batch-normalization, max-pooling, and fully connected layers. The D-miRT model uses two independent CNN components (streams) to process the two sets of feature data and extract higher level features from them independently (Fig. 1B). Given that the two sets of feature data measure qualitatively different aspects of the miRNA TSSs, it is unlikely that there will be any meaningful pattern spanning them. It may confuse the training process if one CNN is used to extract higher-level features from both simultaneously. After the high-level feature extraction steps are complete, the results are merged and analyzed together by a neural network to produce a prediction. The inputs for the two CNN components are a 50 × 2 matrix for peak data representing H3K4me3 and DNase-Seq signals in 50 bins of size 20 bp each covering a 1 kbp region; and a 1000 × 5 matrix for the sequence data, representing the one-hot encoding and its conservation score per nucleotide. The sequence and epigenetic marker information are processed independently by the respective CNN components. The outputs of the two CNN components are flattened and concatenated before being processed by a fully connected neural network with three layers. The third layer of this fully connected network is the output layer with softmax activations that predicts the 11 probabilities corresponding to the 11 types of samples. A probability score threshold of 0.8 is applied on the output 11 by 1 vector of 11 probabilities to make predictions (Fig. 1B). If one entry in the output vector has a probability larger than 0.8, there is a TSS in the corresponding bin of the input region. Otherwise, no TSS is in the input region.

The architecture of the two CNN components are similar and was adopted from the 16-layer version of the VGG model^36^. The VGG architecture was first developed for use in the object recognition application of Computer Vision. It was chosen as a template for this method due to its success at the application of object recognition. Following the VGG architecture, each CNN component consists of four CNN blocks with different filter sizes. Each block is made of two convolution layers, a batch normalization layer and a max pooling layer. The convolutional layers are one dimensional, with filter length of three. The number of filters is different for the four CNN blocks within a CNN component. Part of the reason for this is that stacking multiple convolutional layers with a window size of three simulates the effect of a larger window size while also adding an element of implicit regularization. The aforementioned fully connected neural network, which processes the two CNN components, consists of three layers; the first two layers have 4096 nodes and the final output layer has 11 nodes. To circumvent overfitting, 50% dropout is applied after the first two layers of the fully connected network. The activation function used for each convolutional and the fully connected network layers is the Rectified Linear Unit (ReLU) function, with the exception of the output layer of the fully connected network, which uses a softmax activation function to output 11 probabilities. Training took place using a categorical cross entropy loss function^37^, and stochastic gradient descent optimization^37^. The learning rate was set to 0.01 with a momentum of 0.9, and a weight decay of 0.0005.

The samples in each of the seven cell lines were partitioned into 90% training and 10% test samples. Seven cell line-specific models were trained. To prevent overfitting, three measures were taken. First, as mentioned before, 50% dropout was applied after the first two layers of the fully connected neural network. Second, tenfold cross-validation was applied on the training samples. Third, early stopping was done. The training was done with 20 epochs as the model accuracy did not change after that (Supplementary Figure S1A). As the performance evaluation metrics of the model, accuracy, precision, recall and F1-score were considered. Accuracy scores were calculated by the ratio of the truly predicted samples to the total samples. Recall scores were measured by the ratio of true positive samples to the total positive samples; while precision scores indicated how many of the predicted positive samples were true. The F1-score was calculated using the harmonic mean of precision and recall (Supplementary Figure S1B).

### Model variations

To assess the performance of D-miRT with different training data and different models, several model variations were implemented using high resolution data, low-resolution data, with two activation marker features or seven activation marker features, with or without a score threshold. All models were constructed and trained using Keras with the Tensorflow backend. The training was done on a single Tesla V100 (16G) GPU on the university Newton GPU cluster. The time to train the model using the D-miRT architecture was around 2 to 3 h. The training time might be affected by the resource allocation of the GPU cluster.

#### High and low-resolution data

The 1 kbp TSS-containing regions with 50 bins of 20 bp used to train D-miRT were considered high-resolution data. As the low-resolution data, 5 kbp regions containing 50 bins of size 100 bp were considered.

#### Different architectures

D-miRT uses an architecture with two parallel CNN streams independent from each other as described above. We also tested architectures with different parallel streams and different combinations of data types (DNase-seq, H3K4me3, sequence, conservation) in different streams. Moreover, since ten types of positive samples and one type of negative samples are used to train D-miRT, which results in much more positive samples, we also train a D-miRT model with 10% positives randomly chosen together with all negative samples.

#### Two and seven activation markers

In the D-miRT model, DNase-seq and H3K4me3 are the only epigenetic markers used as input to the first CNN component (Fig. 1B). Along with them, five other histone markers; H3K27ac, H3K27me3, H3K36me3, H3K4me1 and H3K9me3 are used in the model variations with seven marker features. The H3K4me3, H3K27ac, H3K4me1, H3K9me3 and H3K27me3 signals are also associated with TSSs, in which H3K27ac is an active marker, H3K4me3 and H3K4me1 can be active or poised markers, and H3K9me3 and H3K27me3 are markers for transcription repression. H3K36me3 is an active marker of gene transcription at gene bodies. The five histone marks are appended to the original DNase-seq and H3K4me3 markers to create a 50 × 7 input matrix as an input to the second CNN component.

#### With or without a score threshold

The D-miRT model outputs an 11 × 1 vector containing the probability distribution for the 11 types of samples. The score threshold corresponds to the minimum prediction probability in the output vector required for a region to be claimed as negative or accepted as positive with the TSS in the corresponding bins. D-miRT uses the threshold 0.8, which means, out of the 11 output, the type with at least 0.8 probability gets the value 1, while others get the value 0. If there is no type with at least 0.8 probability, the input region is not predicted. In case of the model variations without the score threshold, only the type with the highest probability was assigned the value 1.

### Comparison with other known prediction methods in terms of experimental evidence

In order to support D-miRT model predictions with experimental evidence, the distributions of three TSS representative signals, GRO-cap, CAGE and H3K4me3, were used around the D-miRT predicted TSSs located within 50 kb region around the start positions of pre-miRNAs in miRbase 22. These distributions were then compared with two recently published TSS prediction methods, one by Hua et al. and the other called PROmiRNA^22,24^.

We also compared D-miRT with ADAPT-CAGE, microTSS, and PROmiRNA on HESC cell line, where 72 experimentally determined miRNA TSSs were known based on the microTSS study. ADAPT-CAGE applies machine learning methods to filter CAGE signals and identify TSSs at 1 bp resolution. The input is three CAGE BAM files download from the FANTOM project (CNhs14067, CNhs14068, CNhs13964. We then converted the BAM files to the SAM files and ran ADAPT-CAGE with the following command: *perl ADAPT-CAGE.pl –result_directory_id* = *13523-145F4 –species* = *hg19 –cage_file* = *CNhs14068_13523-145F4.sam –cage_file_type* = *sam –genome_file* = *hg19.fa*. We directly used the predictions of microTSS in its original study as microTSS predicted TSSs. We ran PROmiRNA with the following command: *python test_new_regions.py test_regions test_regions.gff*.

## Results

The deep learning model, D-miRT, aims to identify whether a sequence of DNA contains a TSS by learning features from integrating low-resolution epigenetic markers and high-resolution sequence features. The performance of D-miRT was evaluated in comparison with the designed model variations (Material and Methods). The performance of D-miRT was also analyzed on the task of predicting TSSs specific to the seven cell lines of training data sets. The supporting experimental evidence on D-miRT predicted TSSs was presented in comparison with the approach to predict cell-specific TSSs by Hua et al. and another approach called PROmiRNA^22,24^. Finally, specific areas in the input regions were identified that helped D-miRT learn to identify miRNA TSSs.

### D-miRT performance on validation and test data

D-miRT was trained with 90% of the training data in each of the seven cell lines to generate seven cell-specific models. The tenfold cross-validation process was performed separately on the seven cell-specific models. The accuracy, precision, recall and F1-score of the D-miRT models all were around 95% on average during the tenfold cross-validation process (Supplementary Table S2). When evaluated on the 10% test data for the respective cell lines, which were not used for training, D-miRT models for the seven cell lines showed around 92–96% of precision, recall and F1 scores on average with the default score threshold (Table 1). The performance of the models was still high without the threshold. We also trained 2-layer models in K562 and GM12878, in which we trained a model to predict whether a region contained a TSS and then trained another model to predict which bin in this region contained a TSS. Tested on the 10% hold-out data, the D-miRT models showed better performance than the two-layer models (Table 1).

**Table 1:** The performance of the cell-specific D-miRT models on the 10% test data.

Cell line	# Pos	# Neg	Accuracy	Precision	Recall	F1-score
A549	7645	742	0.9577 (0.8660)	0.9629 (0.8753)	0.9511 (0.8503)	0.9570 (0.8626)
GM12878	9935	943	0.9329 (0.8081)	0.9473 (0.7971)	0.9215 (0.8129)	0.9342 (0.8049)
GM12878*	9935	943	0.9243 (0.8491)	0.9420 (0.8608)	0.9219 (0.8490)	0.9293 (0.8509)
Hela-S3	9968	960	0.9410 (0.8302)	0.9422 (0.8254)	0.9379 (0.8320)	0.9400 (0.8287)
HepG2	10345	986	0.9418 (0.8381)	0.9509 (0.8316)	0.9332 (0.8444)	0.9420 (0.8379)
hESC	9120	873	0.9450 (0.8425)	0.9511 (0.8620)	0.9447 (0.8229)	0.9479 (0.8420)
K562	8019	762	0.9479 (0.8722)	0.9310 (0.8722)	0.9534 (0.8712)	0.9421 (0.8717)
K562*	8019	762	0.9388 (0.9079)	0.9386 (0.9160)	0.9385 (0.9079)	0.9385 (0.9096)
MCF-7	9259	901	0.9470 (0.8531)	0.9505 (0.8293)	0.9341 (0.8973)	0.9422 (0.8619)

The numbers in parenthesis are those without threshold values. The #Pos and #Neg are the number of positive samples and negative samples used to test the trained cell-specific models. The two rows with “*” next to cell line names are from the two-layer models.

The performance of the seven cell-specific D-miRT models was then compared with different model variations on the 10% test data (Table 2). The model performance scores were not too much affected by the change of resolution of the test data. As expected, all model performance scores dropped when the threshold was removed from consideration. The threshold improved the model performance because a positive sample with a TSS bin was likely to be detected several times while a negative sample was unlikely to have any bin scored high. Also, the consideration of five more activation markers in addition to the two used by D-miRT (DNase-seq and H3K4me3) did not have much impact on the performance. This observation obviates the need for more features than the ones currently used in the D-miRT. Moreover, more streams slightly decreased the performance, likely because more complicated architectures require more time and resources to train the models accurately. In addition, the D-miRT models trained with 10% of positive samples reduced their accuracy because of much smaller training samples used.

**Table 2:** Performance of D-miRT in comparison to the model variations.

Model variations	Accuracy	Precision	Recall	F1-score
Low resolution	0.8643	0.8495	0.8647	0.8570
Low resolution + threshold	0.9324	0.9458	0.9154	0.9304
High resolution	0.8859	0.8864	0.8859	0.8861
**High resolution + threshold (D-miRT)**	**0.9479**	**0.9310**	**0.9534**	**0.9421**
High resolution + more features	0.8622	0.8622	0.8612	0.8617
High resolution + more features + threshold	0.9395	0.9285	0.9435	0.9359
Quad stream	0.8729	0.8734	0.8730	0.8732
Quad stream + threshold	0.9293	0.9297	0.9286	0.9291
Triple (H3K4me3, DNase, sequence + cons)	0.8738	0.8736	0.8740	0.8738
Triple (H3K4me3, DNase, sequence + cons) + threshold	0.9388	0.9386	0.9385	0.9386
Triple (H3K4me3 + DNase, sequence, cons)	0.8698	0.8701	0.8701	0.8701
Triple (H3K4me3 + DNase, sequence, cons) + threshold	0.9376	0.9375	0.9371	0.9373
D-miRT with 10% positives	0.8708	0.8441	0.8317	0.8379
D-miRT with 10% positives + threshold	0.9211	0.9107	0.8907	0.9006

The D-miRT model showed high performance on both cross-validation and test data. Also, the performance comparison of the model variations presented a consistently high performance of the D-miRT architecture for the seven cell lines. Although the consideration of threshold played a part in increasing the model performance, the performance was also high enough without threshold. The performance indifference of the model variations with more epigenetic marker features proves the additional marker features to be redundant in the TSS prediction task.

### D-miRT performance on unseen cell lines

In order to analyze the ability of D-miRT to predict TSSs in unseen cell lines, we trained D-miRT on the samples from six of the seven cell lines and then tested the trained models on the samples of the seventh cell line (Table 3). The model performance on each unseen cell line was almost always better than the corresponding cell-specific models trained in Table 1 with and without the threshold, which was likely due to the much larger number of training data here than those in Table 1. It also suggested that the cell-specific epigenetic markers and their genomic features could already predict the cell-specific TSS usage. The trained D-miRT models captured such relationship and accurately predicted the TSS in unseen cell lines with the cell-specific activation signals. The values of the four performance metrics were consistently high for all cell lines and ranged from 95 to 99% with threshold for different cell lines. Without the prediction threshold, the performance did go down a little bit but was still high enough. The model showed stable high prediction metrics with and without the threshold for each of the unseen cell lines. This indicates that the cell independent features might be more decisive in recognizing a TSS than cell-specific ones and D-miRT could pick up these features.

**Table 3:** The performance of D-miRT models on unseen cell line data

Cell line	# Pos	# Neg	Accuracy	Precision	Recall	F-1 Score
A549	76252	7627	0.9808 (0.9453)	0.9862 (0.9558)	0.9770 (0.9390)	0.9816 (0.9473)
GM12878	98985	9907	0.9589 (0.8923)	0.9643 (0.9018)	0.9497 (0.8692)	0.9569 (0.8852)
HeLa-S3	99747	9975	0.9717 (0.9199)	0.9714 (0.9237)	0.9820 (0.9063)	0.9767 (0.9149)
HepG2	103381	10340	0.9694 (0.9267)	0.9618 (0.9161)	0.9697 (0.9300)	0.9657 (0.9230)
hESC	90965	9098	0.9597 (0.9025)	0.9742 (0.9288)	0.9461 (0.8819)	0.9600 (0.9048)
K562	88131	8815	0.9823 (0.9485)	0.9859 (0.9498)	0.9800 (0.9371)	0.9830 (0.9434)
MCF-7	92488	9252	0.9710 (0.9295)	0.9674 (0.9226)	0.9785 (0.9379)	0.9729 (0.9302)

The numbers in parenthesis are those without threshold values.

### Experimental support for the D-miRT predicted TSSs in K562 and GM12878

D-miRT’s performance on finding TSSs on the upstream regions of pre-miRNA start locations was analyzed with experimental evidence and then compared with two recently published approaches, one by Hua et al. and the other called PROmiRNA^22,24^. Here, the 50 kbp upstream regions of the pre-miRNA start locations (miRbase 22) were extracted. Within the 50 kb region, every one kbp region window slid by 100 bp was considered as D-miRT input, resulting in 491 input regions for each pre-miRNA. The epigenetic markers, DNase-Seq and H3K4me3, sequences and conservation scores across the regions were extracted and processed for the input to D-miRT. The 20 bp bin locations, which passed the score threshold 0.8, were considered the predicted TSSs of D-miRT. Because of the sliding window approach, a TSS should be predicted multiple times in different bins. Therefore, only the TSSs predicted at least twice were considered as the predicted TSS.

**Figure 2:**
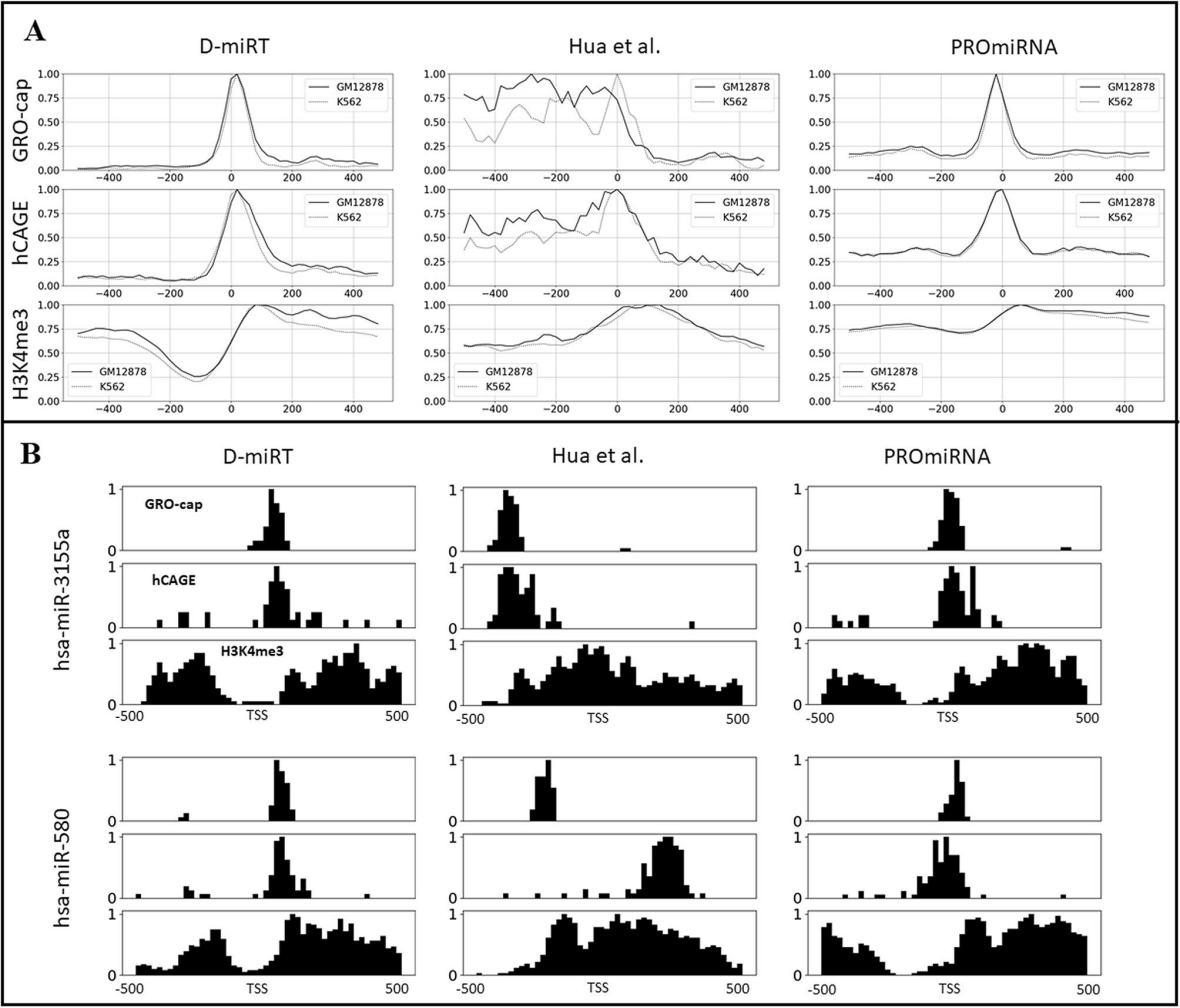
Experimental evidence for D-miRT predicted TSSs. **(A)** The average distribution of GRO-cap, CAGE tags and H3K4me3 in the ± 500 bp regions surrounding the predicted TSSs by D-miRT, Hua et al., 2016 and PROmiRNA in GM12878 and K562 cell lines. **(B)** The distributions of GRO-cap, CAGE tags and H3K4me3 in K562 cell line for two example miRNAs across the ± 500 bp regions surrounding the predicted TSSs by D-miRT, Hua et al., 2016 and PROmiRNA.

To support D-miRT’s performance by experimental evidence, the GRO-cap data from the NCBI GEO database (GSM1480321 and GSM1480323) was considered. GRO-cap is a modified form of GRO-Seq that can identify the 5′ end of cap-protected nascent RNAs^38^. To evaluate the result, the two cell lines K562 and GM12878 were chosen as an additional test case because they are widely used cell lines for TSS prediction and the only cell lines with the GRO-cap data available. GRO-cap profiling in the one kbp regions around the D-miRT predicted TSSs in the cell lines GM12878 and K562 showed that most GRO-cap tags are located at the center of the regions (Fig. 2A).

The GRO-cap, CAGE and H3K4me3 peak coverages around D-miRT predicted TSSs in the 50 kbp region were compared with the predictions made by Hua et al.^24^ and PROmiRNA^22^ (Fig. 2A). Using the weighted sum of the GRO-Cap tags in the 1 kbp region around the predicted TSSs as a score measuring the spread of GRO-Cap tags, predictions by Hua et al. were found to have significantly larger GRO-Cap tag spread than D-miRT predictions (Mann–Whitney test p-value 3.44E-17 in GM12878 and 5.88E-12 in K562) and thus were less consistent with GRO-Cap experiments. The distribution of H3K4me3 and CAGE tags within the ± 500 bp around the D-miRT predicted TSSs, also support the TSSs predicted by D-miRT (Supplementary Table S3). GRO-cap and CAGE tags are known to accumulate around the TSS while H3K4me3 signals are more spread. The distributions of the three signals around PROmiRNA predicted TSSs were more consistent with the distributions around D-miRT predicted TSSs than Hua et al. (Supplementary Table S3).

We also compared the difference between the distribution of the three signals (GRO-Cap, CAGE and H3K4me3) in the 1 kb region surrounding the known TSS and the predicted TSS by the three tools. To calculate this difference, a 50 × 1 vector was generated for each type of signal, which showed the distribution of the signal in 50 bins (20 bp each) around a TSS (known or predicted). The mean absolute difference between the 50 × 1 signal distribution vectors around the known and the predicted TSSs gives a quantitative score about the reliability of the prediction. For both cell lines and all three types of signals, the difference between the vectors around the known TSSs and the D-miRT predicted TSSs was less than those by the Hua et al. and those by PROmiRNA predicted TSSs (Supplementary Table S4, Mann–Whitney test p-value 3.57E-10 and 1.64E-6, respectively). The distribution of GRO-cap, CAGE tags and H3K4me3 signals around the D-miRT predicted TSSs for individual miRNAs show the concentration of GRO-cap and CAGE tags in the vicinity of the predicted TSS bin, while the H3K4me3 signals are high around ± 400 bp and low at the center of the predicted TSS bin (Fig. 2B).

In addition, the robustness of the TSSs predicted by D-miRT was measured by the number of sequence motifs found in the vicinity of the TSSs. The motifs in the ± 10 bp region around the D-miRT predicted TSSs were scanned using FIMO^39^. More than 600 highly significant motifs (p-value < 0.05) were identified in each cell line. The list of top 10 TFs in terms of the number of binding sites within ± 10 bp of the predicted miRNA TSSs included 5 common TFs; MAZ, SALL4, SP1, SP2, VEZF1; for all seven cell lines (Supplementary Table S5). MAZ plays a role in both transcription initiation and termination^40^. SALL4 is known to regulate gene transcription^41^. SP1 and SP2 can activate and repress transcription^42,43^. VEZF1 binds to CT/GC-rich promoter regions and mediates transcription activation^44^. Several motifs indicating the CpG islands around the predicted TSSs were also identified (Supplementary Figure S2). These identified TSS-proximal motifs suggest that the predicted miRNA TSSs are likely to be biologically sound.

### Experimental support for the D-miRT predicted TSSs in hESC

We tested D-miRT on the 72 miRNAs with validated TSSs by Georgakilas et al. in the hESC cell line as well^7^. This was the only cell line shared by our study and the Drosha knockout study by Geogakilas et al. 2014. To predict TSSs of these miRNAs, we started from the 1 kb long windows immediately upstream of the annotated precursors of these 72 miRNAs and slid 100 bp upstream every time to obtain a new window until we reached the upstream 100 kb of these miRNAs. To show that D-miRT is superior to or at least comparable with existing methods, including the CAGE-filtering methods, we compared D-miRT with ADAPT-CAGE, microTSS, and PROmiRNA on these windows. In these comparisons, all windows that overlapping with a given neighborhood (± 50 bp, ± 200 bp, ± 500 bp, or ± 1 kb) of the 72 known TSSs defined by Georgakilas et al. were considered as positives and the remaining windows were negatives. Since the windows overlapping with the 72 known TSSs are essentially 72 positives, it may make more sense to consider to have only 72 positives and have the number of negatives unchanged (Criteria I). On the other hand, we considered all windows overlapping with the neighborhood of the 72 known TSSs as independent positives and kept the negatives the same (Criteria II). The difference between the two criteria was that multiple predicted positives in the neighborhood of a true TSS were considered as the same prediction, although the positive and negative windows were the same in both criteria.

We found that D-miRT predicted at least one TSS in the neighborhood of the 72 true TSSs, even when the neighborhood size considered was 50 bp (Supplementary Table S6). From the smaller neighborhood size (50 bp) to the larger ones (1 kb), about 83% to 61% of bins in the corresponding neighborhood of true TSSs were predicted as TSSs (the corresponding numbers in the parentheses in Supplementary Table S6). On the other hand, ADAPT-CAGE, microTSS and PROmiRNA predicted TSSs were within about 95%, 60%, and 78% of the neighborhood of the 72 true TSSs, respectively.

**Figure 3:**
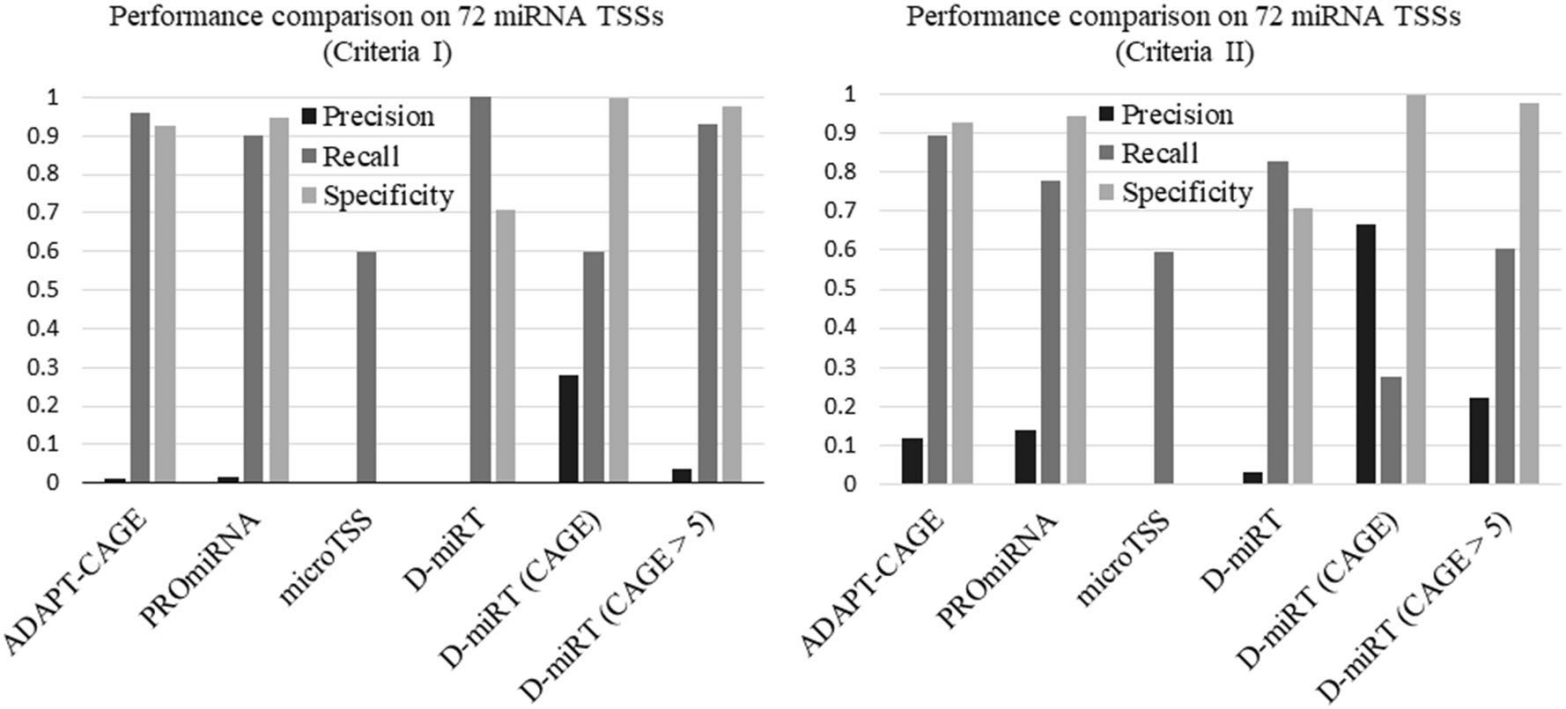
Performance comparison of ADAPT-CAGE, PROmiRNA, microTSS, D-miRT and the two versions of D-miRT with CAGE filtering on their predictions of 72 miRNA TSSs with ± 50 bp neighborhood. Here the performances were shown using two definition criteria of the positives.

Despite the higher recall and sensitivity, D-miRT had the worst precision compared with ADAPT-CAGE and PROmiRNA (Fig. 3). This is likely because other methods essentially filter all windows with low CAGE signals in advance and apply machine learning methods to predict whether the remaining windows are likely to contain TSSs. In other words, we hypothesized that it is not these methods but the CAGE filtering achieving higher precisions. To validate this hypothesis, we overlapped the predicted positive windows by D-miRT with the CAGE peaks defined by FANTOM 5. We found that the resulted predictions indeed had much higher precisions, although the recall was significantly decreased (D-miRT (CAGE)). To have a higher recall and precision, we simply considered the predicted positive windows by D-miRT with at least 5 CAGE signals in the ± 100 bp neighborhood of the corresponding predicted bin as positive windows, which we called D-miRT (CAGE > 5). The CAGE signal was calculated based on the same BAM files used by ADAPT-CAGE. We found that the filtered D-miRT predictions, which we called D-miRT (CAGE > 5), indeed had a superior or at least comparable performance compared with all other methods under both criteria of the defined positives and negatives (Fig. 3).

In Summary, D-miRT is a valuable method for miRNA TSS predictions, especially when there is no CAGE data available. With available CAGE data in a cell line or a cell type, the simply-applied CAGE filtering methods, D-miRT (CAGE > 5) is at least comparably with other CAGE-filtering methods. By combining CAGE signals into the training of the D-miRT model may thus further improve the precision and recall of the miRNA TSS prediction here.

### Features learned by D-miRT

**Figure 4:**
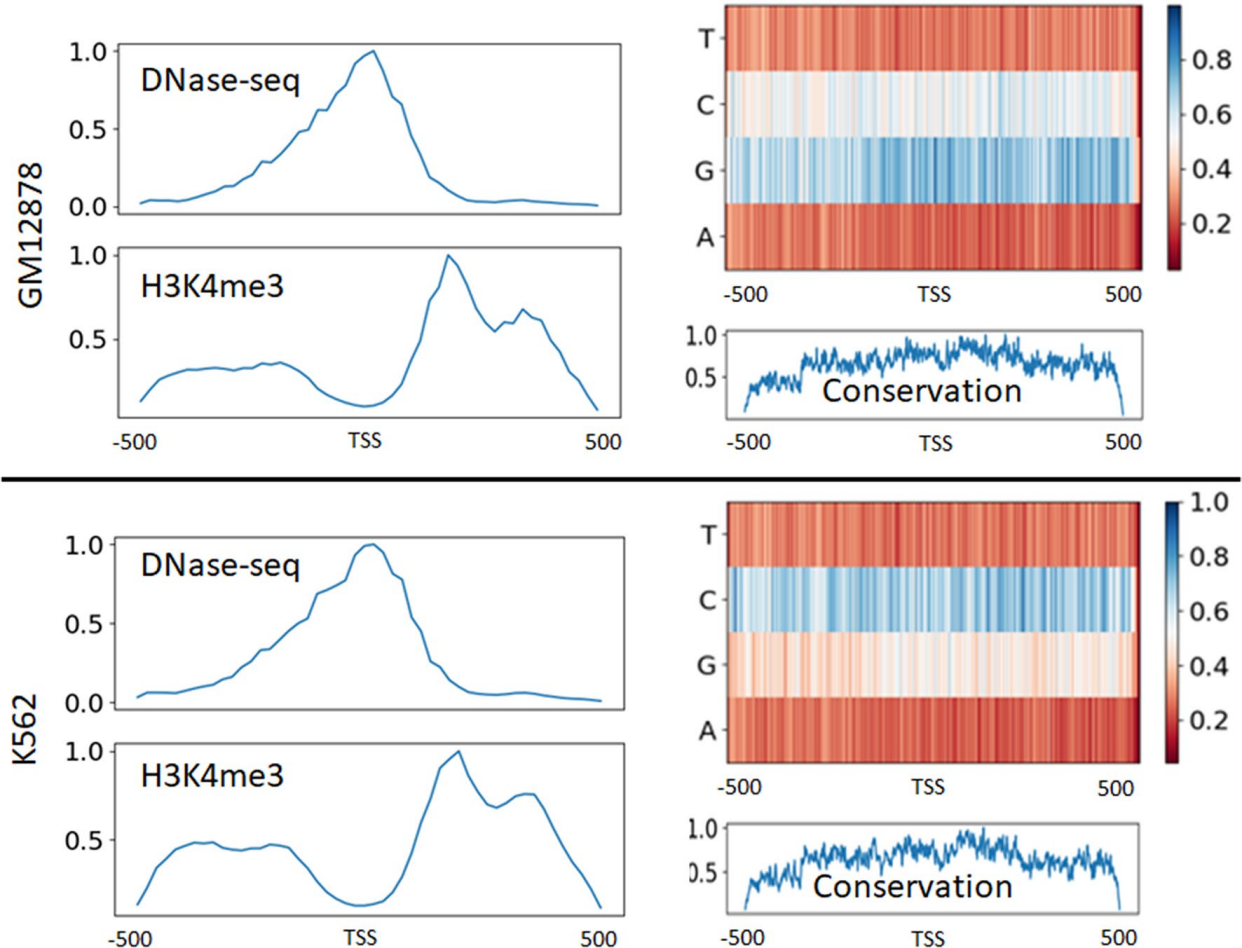
Average activation of CNN Block 4 in terms of the change of the DNase-seq, H3K4me3, sequence (A, G, C, T) and conservation values in different locations within 1 kbp around the D-miRT predicted TSSs. The activations are shown for GM12878 and K562. The heatmap was created using python V3.7 (https://www.python.org/).

In order to identify the localized impacts of the epigenetic markers and the sequence features on the classification decision, the class activation mapping technique was adopted^45^. This technique helped find exact positions of the input region the model considers important in different inputs. In both CNN components of D-miRT, after the last CNN layer of each CNN block, a 1D average pooling layer was added with a window size spanning the entire output. This converted the output from a C x L matrix to a C × 1 column vector, which was later transposed into a 1 × C row vector and multiplied by the CNN output to obtain a 1 × L row vector representing the average activation at each position in the output. The average activation value after each CNN block provided insight into which portions of the input the model was learning to focus on when it was processed by successive layers. This whole process was carried out using “iNNvestigate” toolbox^46^. The average activation of the last CNN block (block 4) was examined for class activation mapping (Fig. 4). The DNase-seq, H3K4me3, sequence and conservation scores were examined. Upon reaching the last CNN block of the first stream, for DNase-seq features, the network's attention was primarily focused on the center of the 1 kbp input, while the focus area was more spread throughout the input region for H3K4me3. The patterns agree with the known facts that the DNase-seq signals get concentrated in the vicinity of the TSS while the H3K4me3 signals are low at the TSS location and more spread along with the upstream and downstream of the TSS. Among the sequence features, changes of C and G nucleotides had the most impact on the activation of block 4. Overall, changes of A and T nucleotides did not contribute as much to the activation of the last CNN block, as the changes of C and G. This observation agrees with the known fact that the surrounding area of a TSS is rich in CpG islands^47^. The changes in the conservation scores near the TSS and downstream of the TSS triggered the activation of block 4 than the upstream, indicating the higher contributions of the conservation features across the gene body.

### Sequence features were the most important feature to D-miRT

The contribution of the four types of inputs, DNase-seq, H3K4me3, sequence and conservation, in training the D-miRT model was analyzed by input omission technique. Four different models were trained with one of the four types of inputs removed from the training data each time. The accuracy, precision, recall and F1-score of the four models were measured to check the effect of the removed feature. The four metric scores for the two activation marker inputs (DNase-seq and H3K4me3) were similar, with DNase-seq showing higher importance, which may be due to the narrower peaks of DNase-seq compared with HK4me3 peaks (Table 4). The removal of the sequence input affected the performance of the model the most. This indicates the importance of the sequence-specific features in TSS identification. Eliminating conservation input did not affect the model performance to the same degree, suggesting that not all neighboring regions around TSSs are conserved, and conservation only helps the identification of certain TSSs (Table 4).

**Table 4:** The impact of input features on D-miRT training.

Data type	Accuracy	Precision	Recall	F1-score
DNase-seq	0.8627	0.8774	0.8350	0.8557
H3K4me3	0.8813	0.8878	0.8646	0.8761
Sequence	0.7612	0.8052	0.7570	0.7804
Conservation	0.9174	0.9111	0.9258	0.9184

To investigate the contribution conservation score input, the conservation input was removed from the D-miRT design and the GRO-cap, CAGE and H3K4me3 patterns around the predicted TSS were compared with the corresponding signal patterns by the original D-miRT model. Although not obvious, the GRO-cap, CAGE and H3K4me3 patterns looked smoother around the TSSs predicted by the model with the conservation input than the model without the conservation input. The activation of the last CNN block (similar to the previous subsection) with the change in the sequence features became clearer when the conservation input was used in the model (Supplementary Figure S3). All these results suggested that although the contribution of the conservation feature in TSS identification may be less than other features, it actually helped the model to understand other inputs by filtering extraneous noise.

## Discussion

Condition-specific miRNA TSS prediction is critical to the understanding of miRNA gene regulation. However, predictions by existing methods were often either in very low-resolution or not condition-specific. To overcome these challenges, we presented the two-stream CNN-based deep learning model D-miRT for condition-specific miRNA TSS prediction. Tested by cross-validation and on independent data, we showed the good accuracy of the D-miRT model. Compared with two existing methods, D-miRT also showed superior performance.

The motivation of the development of D-miRT is threefold. First, most current methods use SVM-based algorithms to identify miRNA TSSs. They often take in manually curated features. However, features such as DNA sequence motifs are not completely annotated^48,49^. The incompletely or inconsistently defined feature sets for SVMs have potential negative consequences on the prediction accuracy and consistency, whereas, deep learning-based methods are able to extract features automatically. Without the necessity of manual feature curation, deep learning models are more suitable for miRNA TSS identification. Second, the two-stream CNN model is a natural fit for the problem since it is specifically designed to integrate features at different resolutions. Last, although deep learning strategies have achieved a large degree of success when applied to bioinformatics problems including miRNA target prediction and pre-miRNA prediction, they have not been applied to study condition-specific miRNA TSS prediction so far. To our knowledge, this is the first computational approach to flexibly integrate multiple types of features for miRNA TSS predictions.

Large-scale CAGE experimental data and other genomics/epigenomics data enabled the development of D-miRT. As shown in the Results section, D-miRT models trained by data from seven cell lines separately were able to achieve sufficiently high accuracy on the test and validation data. D-miRT design was robust enough to perform highly with both high and low resolution data. D-miRT outperformed other possible model variations. For unseen TSSs, D-miRT performed equally well as it did with the test data. The D-miRT predicted TSSs in the 50 kbp upstream of pre-miRNAs were supported by experiential evidences like GRO-cap, CAGE and H3K4me3 signals. The predicted TSSs were also rich in motifs representing CpG islands. Application of the input perturbation technique showed the importance of specific DNase-seq and H3K4me3 patterns, C and G nucleotides abundance near the TSS. The prediction resolution of D-miRT is currently set to 20 bp regions, which is among the highest resolutions compared with the state-of-art TSS annotation tools that use the same types of data as inputs. Note that methods such as FANTOM5 and Hua et al., 2016 achieved a single bp resolution by post-processing the predictions through the identification of the nucleotide location with maximal tag count in a predicted region. Besides, the D-miRT framework is flexible and not limited by the current input formats. Given more reliable training and testing data available in the future, the resolution can be further increased. However, current miRNA TSS annotation is still far from complete^26^. There are potentially various types of noises embedded in the training data. The prediction accuracy is also highly dependent on the data quality of the input features for a given experimental condition.

MiRNA transcription is a complex process, and various patterns of TSSs have been reported based on the study of large-scale CAGE experiments^50–52^. Observing the predicted TSS regions, we can analyze both epigenetic marker and sequence feature patterns D-miRT has discovered. However, current understanding of the RNA world is still very limited. The training of D-miRT here is only limited to the TSSs defined as narrow peaks by FANTOM project. Therefore, D-miRT is trained to only predict TSSs supported by narrow-peak patterns but not broad-peak patterns in its current format. One potential future study would be integrating the TSSs defined by broad peaks of FANTOM project to train additional models and combine that for more general miRNA TSS prediction. Applying task-specific training data concerning features at different-level of resolutions, D-miRT can also be extended to model other non-coding RNA TSSs. Recent development of GRO-Seq and PRO-Seq technology has also generated a large amount of data on nascent transcript detection. Future application of D-miRT may also include integrating the GRO-Seq data for training and modeling to improve condition-specific miRNA TSS prediction further.

## Supplementary Information

Supplementary Information.
