# Supplementary material for: A two-stream convolutional neural network for microRNA transcription start site feature integration and identification
Source: Sci Rep. 2021 Mar 11;11:5625. doi: 10.1038/s41598-021-85173-x (PMC7952457; doi:10.1038/s41598-021-85173-x)
Supplement: Supplementary file 1 — Supplementary Information. [file 41598_2021_85173_MOESM1_ESM.docx]

Supplementary Tables and Figures

A two-stream convolutional neural network for microRNA transcription start site feature integration and identification

Mingyu Cha, Hansi Zheng, Amlan Talukder, Clayton Barham, Xiaoman Li, Haiyan Hu

**Table S1:** Training data sources

| Cell line | Type | FID | Lab | chain |
| --- | --- | --- | --- | --- |
| A549 | Dnase-seq | ENCFF716ZOM | H3K4me3 ChIP-seq on A549 cell line treated with 100 nM dexamethasone for 12 hours. | hg38 |
| A549 | H3K4me3 | ENCFF375KMZ | H3K4me3 ChIP-seq on A549 cell line treated with 100 nM dexamethasone for 12 hours. | hg19 |
| HeLa-S3 | Dnase-seq | ENCFF912JKA | John Stamatoyannopoulos, UW | hg38 |
| HeLa-S3 | H3K4me3 | ENCFF871ZRW | John Stamatoyannopoulos, UW | hg19 |
| HepG2 | Dnase-seq | ENCFF591XCX | John Stamatoyannopoulos, UW | hg38 |
| HepG2 | H3K4me3 | ENCFF663ZBD | John Stamatoyannopoulos, UW | hg19 |
| hESC | Dnase-seq | ENCFF571SSA | John Stamatoyannopoulos, UW | hg19 |
| hESC | H3K4me3 | ENCFF285ZJI | Bing Ren, UCSD | hg19 |
| MCF-7 | Dnase-seq | ENCFF916WEW | John Stamatoyannopoulos, UW | hg19 |
| MCF-7 | H3K4me3 | ENCFF091CNO | John Stamatoyannopoulos, UW | hg19 |
| GM12878 | Dnase-seq | ENCFF775ZJX | John Stamatoyannopoulos, UW | hg19 |
| GM12878 | H3K4me3 | ENCFF342CXS | John Stamatoyannopoulos, UW | hg19 |
| GM12878 | GRO-cap | GSM1480323 | Leighton James Core, Cornell University | hg19 |
| K562 | Dnase-seq | ENCFF441RET | John Stamatoyannopoulos, UW | hg19 |
| K562 | H3K4me3 | ENCFF915MJO | John Stamatoyannopoulos, UW | hg19 |
| K562 | GRO-cap | GSM1480321 | Leighton James Core, Cornell University | hg19 |

| Validation # | Accuracy | Precision | Recall | F1-score |
| --- | --- | --- | --- | --- |
| 1 | 0.9478 | 0.9195 | 0.9716 | 0.9448 |
| 2 | 0.9483 | 0.9396 | 0.9675 | 0.9534 |
| 3 | 0.9496 | 0.9362 | 0.9571 | 0.9465 |
| 4 | 0.9440 | 0.9346 | 0.9509 | 0.9427 |
| 5 | 0.9469 | 0.9645 | 0.9299 | 0.9468 |
| 6 | 0.9460 | 0.9536 | 0.9409 | 0.9472 |
| 7 | 0.9462 | 0.9480 | 0.9373 | 0.9426 |
| 8 | 0.9526 | 0.9592 | 0.9438 | 0.9515 |
| 9 | 0.9460 | 0.9394 | 0.9578 | 0.9485 |
| 10 | 0.9498 | 0.9549 | 0.9368 | 0.9458 |

**Table S2:** 10-fold cross validation accuracy

**Table S3:** Mann-Whitney U test p-values supporting that the three signals are significantly concentrated around D-miRT predicted TSSs compared to Hua et al and PROmiRNA predicted TSSs.

|  | Cell line | Region around TSS | CAGE | GRO-cap | H3K4me3 |
| --- | --- | --- | --- | --- | --- |
| D-miRT vs Hua et al | GM12878 | 200 bp | 3.31E-14 | 3.38E-17 | 4.28E-20 |
|  |  | 500 bp | 5.39E-19 | 3.44E-17 | 1.07E-16 |
|  | K562 | 200 bp | 7.72E-12 | 3.10E-07 | 1.62E-28 |
|  |  | 500 bp | 6.73E-25 | 5.88E-12 | 9.64E-20 |
| D-miRT vs PROmiRNA | GM12878 | 200 bp | 2.63E-17 | 5.44E-18 | 2.09E-23 |
|  |  | 500 bp | 2.74E-18 | 7.43E-17 | 4.34E-23 |
|  | K562 | 200 bp | 3.88E-16 | 2.24E-09 | 6.76E-25 |
|  |  | 500 bp | 1.35E-21 | 5.55E-12 | 1.86E-20 |

**Table S4:** The mean absolute distances between the highest peaks around the known and predicted TSSs by the three tools.

|  |  | GRO | CAGE | H3K4me3 |
| --- | --- | --- | --- | --- |
| GM12878 | PROmiRNA | 0.20 | 0.22 | 0.14 |
|  | Hua et al | 0.35 | 0.43 | 0.23 |
|  | D-miRT | 0.16 | 0.11 | 0.13 |
| K562 | PROmiRNA | 0.22 | 0.23 | 0.13 |
|  | Hua et al | 0.33 | 0.35 | 0.23 |
|  | D-miRT | 0.11 | 0.10 | 0.07 |

**Table S5:** Top common TFs in terms the number of binding sites in the ±10 bp around the D-miRT predicted miRNA TSSs in seven cell lines. The number of TF motif binding sites TF motif is shown for the seven cell lines. The last column shows the probability that a random segment of 20 bp long contains the binding site of the corresponding motif. The p-value of the observed number of occurrences of these motifs in the 20 bp neighbor of the predicted TSSs is shown in the parenthesis based on binomial testing

|  | A549 | GM12878 | HES | HelaS3 | HepG2 | K562 | MCF7 | Random chance |
| --- | --- | --- | --- | --- | --- | --- | --- | --- |
| MAZ | 74 (8.33e-15) | 56 (6.22e-15) | 79 (8.66e-15) | 72 (6.44e-15) | 75 (2.64e-14) | 105 (7.33e-15) | 95 (1.02e-14) | 0.019 |
| SALL4 | 64 (6.66e-15) | 51 (4.88e-15) | 80 (7.22e-15) | 57 (5.33e-15) | 67 (9.66e-15) | 86 (7.33e-15) | 74 (7.33e-15) | 0.015 |
| SP1 | 64 (5.00e-15) | 46 (3.77e-15) | 66 (5.44e-15) | 47 (3.89e-15) | 77 (6.55e-15) | 89 (5.55e-15) | 73 (6.66e-15) | 0.012 |
| SP2 | 64 (5.77e-15) | 46 (4.33e-15) | 73 (6.11e-15) | 62 (4.66e-15) | 72 (7.77e-15) | 93 (6.44e-15) | 79 (6.66e-15) | 0.013 |
| VEZF1 | 52 (5.00e-15) | 58 (3.77e-15) | 57 (5.44e-15) | 57 (3.89e-15) | 55 (6.55e-15) | 94 (5.55e-15) | 83 (5.55e-15) | 0.012 |

**Table S6:** The performance comparison among D-miRT, ADAPT-CAGE, PROmiRNA and microTSS. The performance is shown considering 50 bp, 200 bp, 500 bp and 1000 bp neighborhood regions around the 72 miRNA TSSs provided in microTSS. The numbers outside the parenthesis represent the performance considering all the windows overlapped with one miRNA TSS neighborhood only once. The numbers inside the parenthesis show the performance counting the overlapped windows as separate positives.

| TSS region | Tool | Positives | Negatives | Precision | Recall | Specificity | TP | TN | FP | FN |
| --- | --- | --- | --- | --- | --- | --- | --- | --- | --- | --- |
| 50 | ADAPT-CAGE | 72 (792) | 71208 (71208) | 0.0129 (0.1181) | 0.9583 (0.8939) | 0.9257 (0.9257) | 69 (708) | 65920 (65920) | 5288 (5288) | 3 (84) |
| 50 | PROmiRNA | 72 (792) | 71208 (71208) | 0.0166 (0.138) | 0.9028 (0.7803) | 0.9458 (0.9458) | 65 (618) | 67347 (67347) | 3861 (3861) | 7 (174) |
| 50 | microTSS | 72 (72) | NA (NA) | NA (NA) | 0.5972 (0.5972) | NA (NA) | 43 (43) | NA (NA) | NA (NA) | 29 (29) |
| 50 | D-miRT | 72 (792) | 71208 (71208) | 0.0034 (0.0304) | 1 (0.827) | 0.7063 (0.7063) | 72 (655) | 50297 (50297) | 20911 (20911) | 0 (137) |
| 50 | D-miRT (CAGE > 5) | 72 (792) | 71208 (71208) | 0.0381 (0.2198) | 0.9306 (0.6023) | 0.9762 (0.9762) | 67 (477) | 69515 (69515) | 1693 (1693) | 5 (315) |
| 50 | D-miRT (CAGE) | 72 (792) | 71208 (71208) | 0.2792 (0.6647) | 0.5972 (0.2778) | 0.9984 (0.9984) | 43 (220) | 71097 (71097) | 111 (111) | 29 (572) |
| 200 | ADAPT-CAGE | 72 (1008) | 70992 (70992) | 0.0132 (0.1393) | 0.9583 (0.8284) | 0.9273 (0.9273) | 69 (835) | 65831 (65831) | 5161 (5161) | 3 (173) |
| 200 | PROmiRNA | 72 (1008) | 70992 (70992) | 0.0174 (0.1666) | 0.9167 (0.7401) | 0.9474 (0.9474) | 66 (746) | 67259 (67259) | 3733 (3733) | 6 (262) |
| 200 | microTSS | 72 (72) | NA (NA) | NA (NA) | 0.8889 (0.8889) | NA (NA) | 64 (64) | NA (NA) | NA (NA) | 8 (8) |
| 200 | D-miRT | 72 (1008) | 70992 (70992) | 0.0035 (0.0367) | 1 (0.7857) | 0.7074 (0.7074) | 72 (792) | 50218 (50218) | 20774 (20774) | 0 (216) |
| 200 | D-miRT (CAGE > 5) | 72 (1008) | 70992 (70992) | 0.0413 (0.2613) | 0.9583 (0.5625) | 0.9774 (0.9774) | 69 (567) | 69389 (69389) | 1603 (1603) | 3 (441) |
| 200 | D-miRT (CAGE) | 72 (1008) | 70992 (70992) | 0.3359 (0.7372) | 0.6111 (0.2421) | 0.9988 (0.9988) | 44 (244) | 70905 (70905) | 87 (87) | 28 (764) |
| 500 | ADAPT-CAGE | 72 (1440) | 70560 (70560) | 0.0138 (0.1758) | 0.9583 (0.7319) | 0.93 (0.93) | 69 (1054) | 65618 (65618) | 4942 (4942) | 3 (386) |
| 500 | PROmiRNA | 72 (1440) | 70560 (70560) | 0.0183 (0.2076) | 0.9167 (0.6458) | 0.9497 (0.9497) | 66 (930) | 67011 (67011) | 3549 (3549) | 6 (510) |
| 500 | microTSS | 72 (72) | NA (NA) | NA (NA) | 0.9028 (0.9028) | NA (NA) | 65 (65) | NA (NA) | NA (NA) | 7 (7) |
| 500 | D-miRT | 72 (1440) | 70560 (70560) | 0.0035 (0.0468) | 1 (0.7014) | 0.7087 (0.7087) | 72 (1010) | 50004 (50004) | 20556 (20556) | 0 (430) |
| 500 | D-miRT (CAGE > 5) | 72 (1440) | 70560 (70560) | 0.0436 (0.3023) | 0.9583 (0.4556) | 0.9785 (0.9785) | 69 (656) | 69046 (69046) | 1514 (1514) | 3 (784) |
| 500 | D-miRT (CAGE) | 72 (1440) | 70560 (70560) | 0.3947 (0.7915) | 0.625 (0.1819) | 0.999 (0.999) | 45 (262) | 70491 (70491) | 69 (69) | 27 (1178) |
| 1000 | ADAPT-CAGE | 72 (2160) | 69840 (69840) | 0.0145 (0.2176) | 0.9583 (0.6042) | 0.9328 (0.9328) | 69 (1305) | 65149 (65149) | 4691 (4691) | 3 (855) |
| 1000 | PROmiRNA | 72 (2160) | 69840 (69840) | 0.0195 (0.2588) | 0.9167 (0.5366) | 0.9525 (0.9525) | 66 (1159) | 66520 (66520) | 3320 (3320) | 6 (1001) |
| 1000 | microTSS | 72 (72) | NA (NA) | NA (NA) | 0.9444 (0.9444) | NA (NA) | 68 (68) | NA (NA) | NA (NA) | 4 (4) |
| 1000 | D-miRT | 72 (2160) | 69840 (69840) | 0.0035 (0.0609) | 1 (0.6083) | 0.71 (0.71) | 72 (1314) | 49588 (49588) | 20252 (20252) | 0 (846) |
| 1000 | D-miRT (CAGE > 5) | 72 (2160) | 69840 (69840) | 0.0449 (0.323) | 0.9583 (0.3245) | 0.979 (0.979) | 69 (701) | 68371 (68371) | 1469 (1469) | 3 (1459) |
| 1000 | D-miRT (CAGE) | 72 (2160) | 69840 (69840) | 0.4018 (0.7976) | 0.625 (0.1222) | 0.999 (0.999) | 45 (264) | 69773 (69773) | 67 (67) | 27 (1896) |


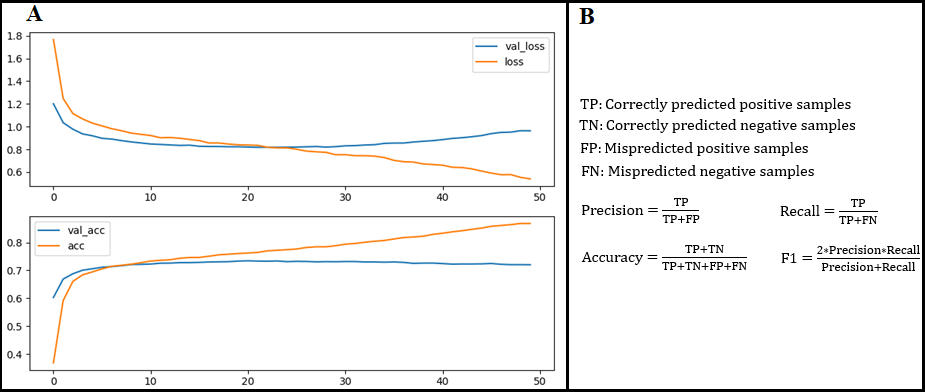


**Figure S1. (A)** Training performance of D-miRT for A549. **(B)** Explanation of the prediction evaluation metrics


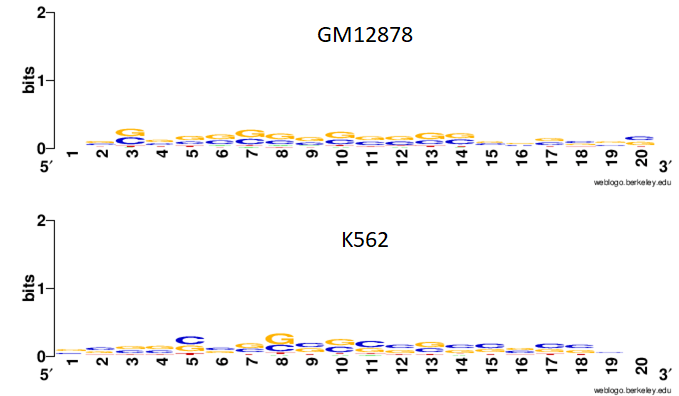


**Figure S2:** Motifs representing CpG islands found in the ±10bp around D-miRT predicted miRNA TSSs.


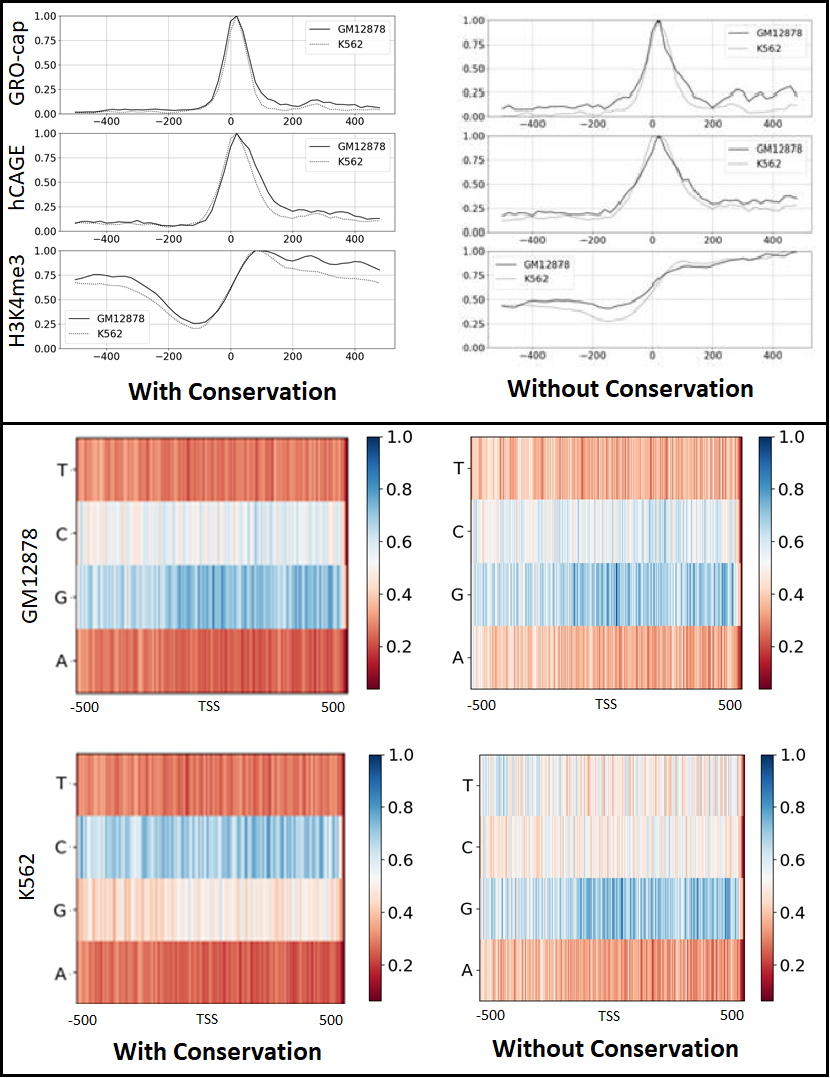


**Figure S3:** (Top) Comparison of the GRO-cap, hCAGE and H3K4me3 patterns around the TSSs predicted by model with conservation and model without conservation. (Bottom) Comparison the activation heatmaps of the last CNN block (block 4) for model with conservation and model without conservation with the changes in sequence input. The heatmap was created using python V3.7 ([https://www.python.org/](https://nam02.safelinks.protection.outlook.com/?url=https%3A%2F%2Fwww.python.org%2F&data=04%7C01%7Camlan%40Knights.ucf.edu%7C24c0012f57ca4c6acb9f08d8b8a91617%7C5b16e18278b3412c919668342689eeb7%7C0%7C0%7C637462383652945670%7CUnknown%7CTWFpbGZsb3d8eyJWIjoiMC4wLjAwMDAiLCJQIjoiV2luMzIiLCJBTiI6Ik1haWwiLCJXVCI6Mn0%3D%7C1000&sdata=FgyybviNEN%2FQNP1jAoqAQdNTzKvq6vdNzvwCF6QBStM%3D&reserved=0)).
